# Capsid protein expression and adeno-associated virus like particles assembly in *Saccharomyces cerevisiae*

**DOI:** 10.1186/1475-2859-11-124

**Published:** 2012-09-11

**Authors:** Ana Backovic, Tiziana Cervelli, Alessandra Salvetti, Lorena Zentilin, Mauro Giacca, Alvaro Galli

**Affiliations:** 1 Laboratorio di Tecnologie Genomiche, Istituto di Fisiologia Clinica, CNR, Pisa, Italy; 2 Scuola Normale Superiore Pisa, Pisa, Italy; 3 Sezione di Biologia e Genetica, Dipartimento di Morfologia Umana e Biologia Applicata, Università Pisa, Pisa, Italy; 4 Molecular Medicine Laboratory, International Centre for Genetic Engineering and Biotechnology (ICGEB), Trieste, Italy

## Abstract

**Background:**

The budding yeast *Saccharomyces cerevisiae* supports replication of many different RNA or DNA viruses (e.g. Tombusviruses or Papillomaviruses) and has provided means for up-scalable, cost- and time-effective production of various virus-like particles (e.g. Human Parvovirus B19 or Rotavirus). We have recently demonstrated that *S. cerevisiae* can form single stranded DNA AAV2 genomes starting from a circular plasmid. In this work, we have investigated the possibility to assemble AAV capsids in yeast.

**Results:**

To do this, at least two out of three AAV structural proteins, VP1 and VP3, have to be simultaneously expressed in yeast cells and their intracellular stoichiometry has to resemble the one found in the particles derived from mammalian or insect cells. This was achieved by stable co-transformation of yeast cells with two plasmids, one expressing VP3 from its natural p40 promoter and the other one primarily expressing VP1 from a modified AAV2 Cap gene under the control of the inducible yeast promoter Gal1. Among various induction strategies we tested, the best one to yield the appropriate VP1:VP3 ratio was 4.5 hour induction in the medium containing 0.5% glucose and 5% galactose. Following such induction, AAV virus like particles (VLPs) were isolated from yeast by two step ultracentrifugation procedure. The transmission electron microscopy analysis revealed that their morphology is similar to the empty capsids produced in human cells.

**Conclusions:**

Taken together, the results show for the first time that yeast can be used to assemble AAV capsid and, therefore, as a genetic system to identify novel cellular factors involved in AAV biology.

## Introduction

Adeno-associated Virus (AAV) of the *Dependovirus* genus is a member of the *Parvoviridae*, a family of small and the simple viruses, whose 4.7 kb single stranded DNA (ss-DNA) is enclosed in a non-enveloped, 20–25 nm icosahedral capsids [1,2]. For more than 25 years, the AAV genome has been thought to contain two genes, *rep* and *cap*, corresponding to two open reading frames (ORFs) and coding for four Rep proteins that regulate replication and three VP proteins (subunits) that form capsids. Rep68 and 78 are transcribed from the p5 promoter and Rep52 and Rep40 from the p19 promoter. The p40 promoter regulates the transcription of the *cap* gene encoding for the VP1, VP2 and VP3 proteins that form the 60 subunit capsids in the proper stoichiometry 1:1:10. This precise VP1, 2 and 3 protein ratio is thought to be the consequence of the alternative splicing required for VP1 expression and the usage of an uncommon ACG site for VP2 translation initiation [3,4]. Only recently, an alternative AAV ORF has been mapped in the *cap* gene and codes for the assembly activating protein (AAP) that promotes capsid assembly in 293 T cells [5]. Rep68 and 78 participate in the AAV DNA replication and regulate transcription from AAV promoters and some host-cell promoters; Rep40 and 52 are involved in the generation and accumulation of single–stranded viral genomes from double stranded replication intermediates. The coding region of AAV genome is flanked by two 145 nucleotide long inverted terminal repeats (ITRs). The ITRs are the only cis-acting elements necessary for AAV replication, packaging and integration [6]. AAV-based vectors have quickly gained great popularity in gene therapy applications owing to: (i) reduced ethical concerns regarding the nonpathogenic nature and limited viral sequences retained in vectors and (ii) favorable properties, such as high efficiency of transduction of post-mitotic tissues and the long-term persistence of transgene expression. The rising number of AAV vector-based gene therapy trials that require high vector doses, over 10^13^ genome copies (g.c.)/kg of body weight [7], resulted in a variety of currently existing systems for rAAV vectors’ production, based on mammalian and insect cell-factories. In view of developing a better rAAV production technology and creating a simple system for revealing still unknown aspects of AAV life cycle, we have recently established a novel, *Saccharomyces cerevisiae* - based recombinant system for ssDNA AAV2 genome formation from the circular vectors [8]. To date, a great number of simple, single subunit virus/nucleocapsid-like particles (VLPs/NLPs) has been efficiently produced in yeast that therefore has a notable value in vaccine production technologies. Apart from offering low-cost and easy to scale-up production, the benefit of this microbial cell-factory is its intracellular environment, highly suitable for the most of metazoan posttranslational processing events, which are a prerequisite for complex multimeric protein interactions [9]. Recently, Rotavirus-like particles were assembled and extracted from *S. cerevisiae* cells [10]. To further explore usefulness of this microbial host, this work describes the permissiveness of *S. cerevisiae* intracellular environment to assembling of AAV type 2 capsids. With the purpose of finding the best expression system to assemble AAV VLPs in yeast cells, we expressed the three capsid proteins from their natural p40 promoter or from yeast promoters, in combination with, or without, plasmid expressing Rep proteins. Finally, the work demonstrates that AAV VLPs morphologically similar to those ones from mammalian or insect cells, can be assembled in the budding yeast *Saccharomyces cerevisiae*.

## Materials and methods

### Plasmids construction

All AAV-2 sequences in the following constructs derive from pSub201 plasmid which contains complete wild-type AAV-2 genome [11]. YEplac181 [12]}, pYES2 (Invitrogen) and pGAD424 (Clontech) are vectors with LEU2 or URA3 genes for yeast selection. To make the plasmid YEplac.p40Cap construct, unmodified AAV2 Cap gene was cut out from pSub201 and cloned in YEplac181 vector, using *Sal*I and *Xba*I sites. For construction of the YEplacRepCap plasmid, the AAV2 genome without ITRs was cut out from pSub201 and cloned in the YEplac181 vector using *Xba*I restriction site. pYESIntronCap construct was made by cutting out AAV2 Intron + Cap sequence (without p40 promoter) from pSub201 and cloning this fragment in the multiple cloning site of pYES2 vector, using *Hind*III and *Xba*I restriction sites. To make the pYESCap plasmid, VP1 cDNA (map position 2203–4410 in wild-type AAV2 genome in the database, GenBank accession no. AF043303.1) was amplified by PCR from pSub201 using following primers:

5' - ctt ATGGCTGCCGATGGTTATCTT-3' and 5' - ctggTTACAGATTACGAGTCAGGTA −3.'

The sequence corresponding to the wild-type AAV2 genome is capitalized. The pYESVP1KM plasmid was constructed by cloning modified VP1 cDNA under Gal1 promoter. The VP1 cDNA was PCR amplified from pSub201vecotr using following primers:

5'-ca**ggatcc***aaaca*ATGGCTGCCGACGGTTATCTACCCGATTGGCTC-3' and

5'-cc**ggatcc**TTACAGATTACGAGTCAGGTATCTGGTGC-3'.

*Bam*HI sites (at each primer end, underlined and bold letters) were introduced outside the VP1 expression cassette for cloning in pYES2 expression plasmid. Beside *Bam*HI restriction site, upper primer also included 5 nucleotide long yeast Kozak sequence (italicized letters) upstream to the VP1 ATG and three modifications (underlined letters) relative to the genuine VP sequence in the database. The sequence corresponding to the wt VP ORF is capitalized. After direct cloning in pYES2 *Bam*HI site, desired clone contained Kozak sequence upstream to the ATG start site and three mutations with respect to genuine AAV2 sequence. The first T to C mutation at position 11 eliminates an out-of frame ATG codon by creating an ACG triplet instead. The second modification is inactivation of the major AAV splice acceptor site, by substituting T to A at position 21 and A to C at position 24 (numbering is as for the sequence in the database).

### Yeast strain, media

The strain RSY12 (*MAT****a****leu2-3,112 his3-11,15 URA3::HIS3*) has a complete deletion of URA3 gene which was replaced with the HIS3 gene [13]. Complete (YPAD) and synthetic complete (SC) mediums were prepared according to standard protocols. Yeast was cultivated aerobically in flasks at 30°C under constant orbital shaking (180 rpm). All yeast cultures were started from a small inoculation (less than 100 μl per 50 ml of culture medium) of stationary phase yeast cells. After over-night growth, cells were collected at desired points of the growth cycle determined by absorbance at 600 nm (OD_600_): early exponential (log) growth phase of O.D_600_ 0.7-1.0, mid-log phase yeast culture of OD_600_ 3.5- 4.0 and early stationary phase O.D_600_ ~8.

The *cap* expressing plasmids YEplacRepCap, YEplacp40Cap, pYESCap and pYESIntronCap, were transformed in yeast cells either independently or in combination, using the standard high-efficiency, lithium chloride based method, using single-stranded DNA as a carrier [14]. Single transformants were selected onto SC-uracil (SC-URA) or SC-leucine (SC-LEU) plates while double transformants were selected onto SC-uracil-leucine (SC-URA-LEU) plates. Monoclonal cultures were repeatedly grown under selection either in the solid or in the corresponding liquid medium. The AAV protein expression was induced with either 5% galactose or different glucose + galactose concentrations as stated in the results.

### Protein extraction

For analysis of AAV proteins by gel electrophoresis, we set up the novel protein extraction method named “Optimized post-alkaline” protein extraction, which consists of two cycles of extraction [8]. Briefly, aliquots corresponding to 100–200 × 10^6^ cells were subjected to the first round of extraction by the method published by Kushnirov [15] with a small variation in the extraction buffer (50 mM TrisHCl pH6.8, 5% glycerol, 3% SDS, 5% β-mercaptoethanol) [15]. After centrifugation, the first protein extract named “extract 1” was ready for further analysis. The pellet was resuspended in harsh RIPA buffer (500 mM NaCl, 50 mM Tris–HCl, 1 mM EDTA, 1% Triton, 1% Deoxycholate (DOC), 1% SDS) and protein solubilization further assisted by sonication. After eliminating the cellular impurities, the resulting proteins fraction was named “extract 2”. Extracts 1 and 2 were analyzed by 10% SDS-PAGE polyacrylamide gel electrophoresis. Protein expression was analyzed by Western blot assay using previously described monoclonal antibodies: anti-Rep monoclonal mAb 303.9, anti-VP1, 2 mAb A69, anti-VP1, 2, 3 mAb B1 [16][17] and anti-3 phosphoglycerate kinase (3PGK, Molecular Probes) as loading control. Like in other systems [18], for numeric estimation of VP protein stoichiometry we used band densitometry analysis performed by means of software Scion Image [18].

### AAV VLP extraction

Aliquots corresponding to 25 ml of yeast culture in log-phase of growth (3 × 10^7^ cells/ml) in medium containg 2% glucose were diluted 10 fold and re-grown on glucose for another 12–16 h until reached OD_600_ 4 (mid-log phase). Then, cells were harvested, washed and inoculated in the induction media (e.g. 0.5% glucose +5% galactose). At different time points, cells were harvested, washed and further analysed. Samples of 10 g of yeast pellet were resuspended in 20 ml of DNS disruption buffer (500 mM NaCl, 10mMTris pH8, 1 mM EDTA, 0.3% NP-40, 0.3% DOC; protease-inhibitor cocktail supplemented) and homogenized by vigorous vortexing at 4°C with 400 nm glass beads. The crude lysate was cleared from cell-debris and undisrupted cells by quick-low speed centrifugation. The cleared lysate was then centrifuged at 20 000 × *g*, for 1 hour at 4°C ; the pellet was subjected to the second extraction performed in the same way as the first one, with additional 10 sonication cycles (15 sec on and 30 sec off; 15 microns amplitude) in order to enhance solubilization of yeast produced AAV VLPs. After sonication, the cell lysate was centrifuged again for 1 hour at 20,000 × *g* at 4°C for elimination of insoluble material. The supernatant was mixed with the first one and subjected to 200,000 × g ultracentrifugation at 4°C through 40% sucrose cushion (40% sucrose in TE-BSA buffer: 10 mM Tris pH8, 1 mM EDTA and 0.01%BSA) for 3 h. After the centrifugation, the pellet was resuspended in 5 ml resuspension buffer (150 mM NaCl, 10 mM Tris–HCl pH 8, 1 mM EDTA, 5 mM MgCl_2_), sonicated and left in agitation for 15 h at 4°C. To improve solubilization of assembly products, the mixture was treated with DNase (Benzonase purity grade I, Novagen) for 30 minuntes at 37°C. Finally, the suspension was cleared by a further centrifugation at 15000 × *g* for 10 minutes at 4°C. The supernatant was loaded on CsCl gradient. CsCl was added to the sample at final density of 1.4 g/cm^3^, and ultracentrifugation was performed at 39,000 rpm for 48 hours, at 18°C, in a SW40-Ti rotor. 12 fractions of 1.371 to 1.430 g/cm^3^ densities (refractometer readings) were collected, dialyzed against PBS buffer, and each fraction was analyzed for VP composition by Western blot.

### Transmission electron microscopy

Fractions were placed on 200-mesh formvar carbon coated copper grids. The sample was stained with five drops of 2.5% (w/v) of uranyl acetate solution and incubated for 2 minutes at room temperature. The excess solution was removed by blotting the edge of each grid onto filter paper and the grid was air dried for 30 minutes. Samples were examined with a Jeol 100 SX transmission electron microscope.

## Results

### Capsid protein expression from the AAV p40 promoter and influence of Rep proteins

**Figure 1:**
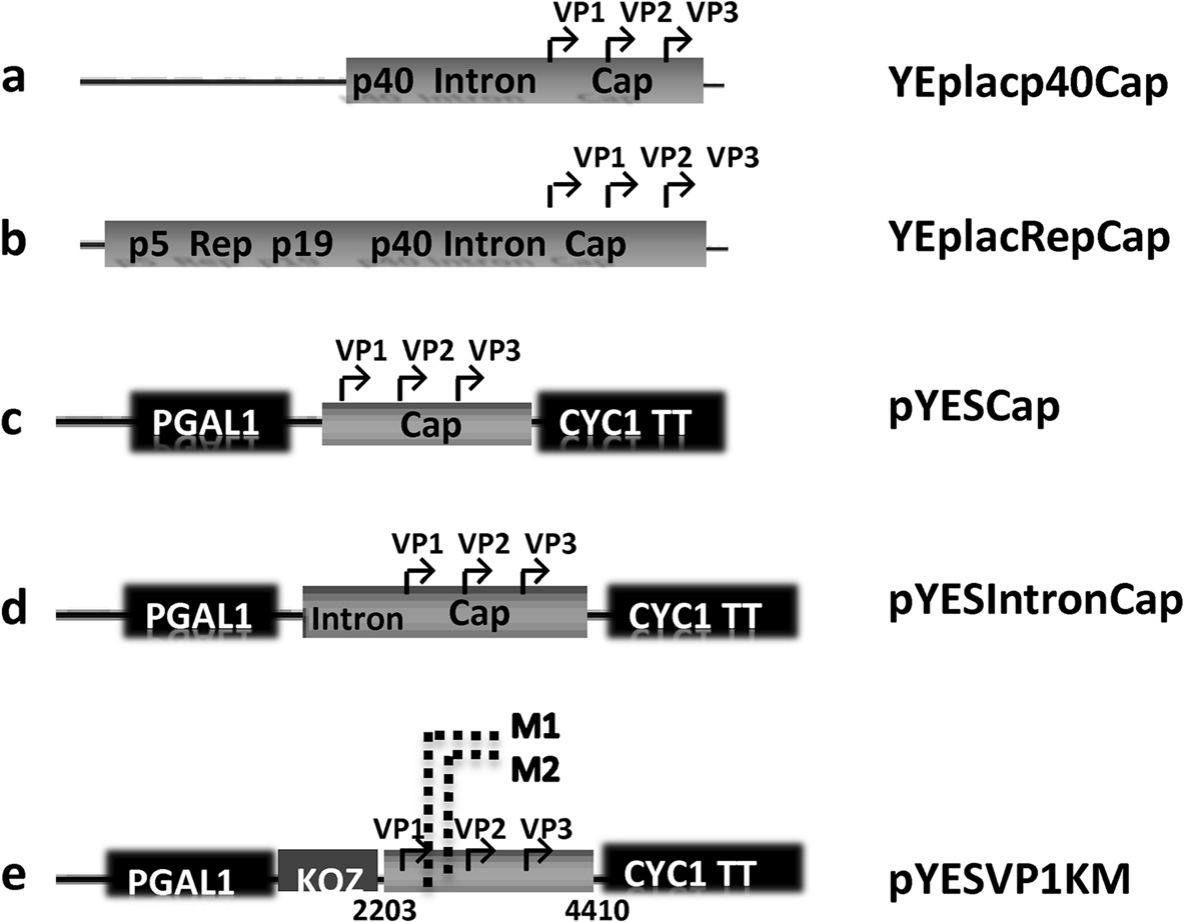
**Schematic representation of expression cassettes constructed in this study.a**) In the plasmid YEplacp40Cap, entire AAV2 cap gene is under control of AAV2 p40 promoter. **b**) the YEplacRepCap plasmid contains *rep* and *cap* gene in wt configuration. **c**) In the vector pYESCap, the entire *cap* gene is under control of Gal1 promoter. **d**) In the plasmid pYESIntronCap, the *cap* gene is placed under control of Gal1 promoter and the AAV2 intron sequence upstream the promoter. **e**) In the vector pYESVP1KM, the VP1 coding sequence, placed under control of Gal1 promoter, was mutated in three sites. The point mutations were made in position 11 to eliminate an out of frame ATG codon (indicated as M1 in the scheme), in position 21 and 24 to inactivate the major AAV splice acceptor site (indicated as M2 in the schemes). A yeast Kozak like sequence was cloned upstream the VP1 start codon.

**Figure 2:**
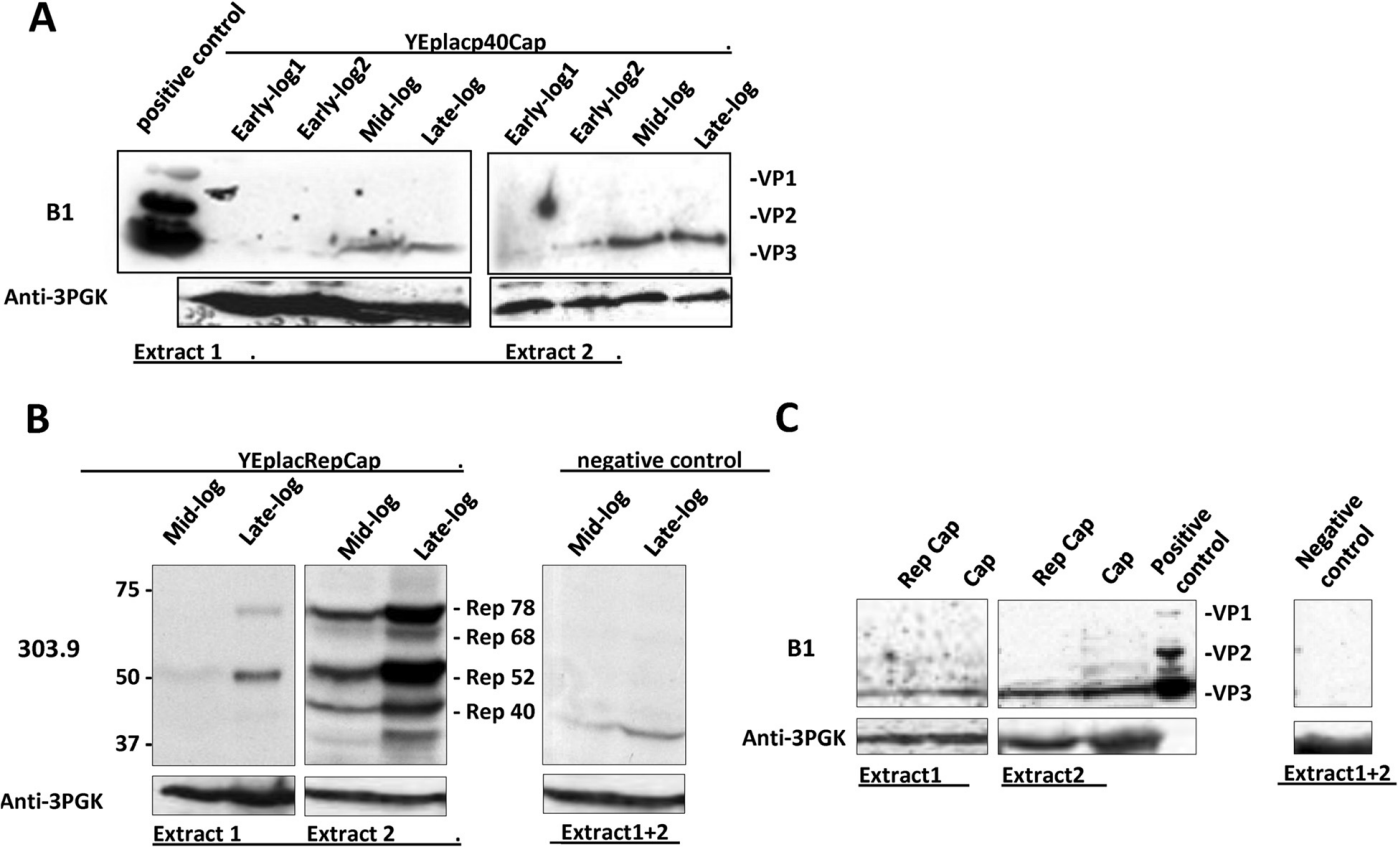
**Expression of AAV2 Cap and Rep proteins from natural promoters.** Transformed RSY12 cells were grown in liquid selective medium until cultures reached different densities determining different growth phases (early → late log) indicated on the top. Equal amounts of total cell lysate, extract 1 and 2 (~50 μg each), obtained from 2 × 10^8^ cells at each phase, were analyzed by Western blot analysis with antibodies indicated on the left: mAb B1 to detect Cap proteins (**A** and **C**), mAb 303.9 to detect Rep proteins (**B**) and mAb Anti-3PGK to detect constitutive yeast protein PGK (Phospho-Glicerate-Kinase). (**A**): VP3 was the only capsid protein detected in the samples from YEplacp40Cap transformed cells;thehighest level was detected in the mid-log phase extracts. (**B**): All four Rep proteins were detected in the samples from YEplacRepCap transformed cells; the highest level was obtained in the late-log phase extracts. (**C**): late-log phase extracts from YEplacRepCap cells were also analyzed for Cap protein expression and compared with late-log phase extracts from YEplacp40Cap cells; only VP3 was detected in both samples. Denatured, 293 T-cell-derived, AAV2 capsids were used as positive control (+ control) for defining VPs (**A**, **C**).

The AAV capsid is composed of 60 subunits with a constant stoichiometry of the proteins VP1, VP2 and VP3 corresponding to 1:1:10 [19,20]. By densitomeric analysis of protein bands resolved by Western blot, the ratio of the AAV structural proteins VP1:VP2:VP3 has been shown to fluctuate from 1:1:8 to 1:1:20 [21]. Moreover, in crude extracts, an average ratio ranging from 1:1:5 to 1:1:20 has been observed [18]. In order to assess the best promoter combination to express the AAV structural proteins at the optimal ratio, we constructed several plasmids carrying yeast constitutive or inducible promoter, or the natural AAV promoter (Figure 1, Table 1). First of all, we studied the expression of the capsid proteins under control of the AAV p40 promoter. We have previously shown that the AAV p5 and p19 promoters can be recognized by the yeast transcriptional machinery [8]. This may imply that also p40 could be functional in this simple eukaryote. We constructed a yeast multi-copy plasmid containing unmodified AAV cap gene with all regulative elements: p40 promoter, intron element and polyadenylation signal (Figure 1 scheme “a”, Table 1). To do this, the entire VP expression cassette located between 1428 and 4495 nucleotides of AAV genome (numbering is as for the sequence under GenBank accession no. AF043303.1) was cloned into YEplac181. The resulting plasmid named YEplacp40Cap has the yeast *2-micron* origin of replication which constantly provides 20–50 copies of the recombinant gene per haploid yeast genome [22]. Plasmid YEplacp40Cap was transformed in the haploid yeast strain RSY12. Growth curves carried out at 30°C under constant shaking of YEplacp40Cap and YEplac181 control plasmid-transformed cells were similar, with doubling time of about 2.7 h, when 2% glucose was used as the carbon source. At 4 different time points, cells were collected and subjected to protein extraction. The first two time points correspond to early exponential/logarithmic (log) growth phase, the third one to mid-log and the fourth one to the late-log phase. Cap protein expression was analyzed by Western blot analysis of total cell lysates at each of these time points (Figure 2A). As shown in the Figure 2A, the only capsid protein detected is VP3. Since the concentration of VP1 and VP2 is expected to be 10 times lower than that of VP3, it is likely that the proteins are present in an amount too low to be detected by western blot. VP3 accumulated with time, following exponential cell growth and biomass accumulation (the maximal amount of protein was extracted from mid-late exponential growth phases). The majority of VP3 protein was found in insoluble fraction from which it was extracted (see materials and methods). This fraction of the total cellular proteins was designated as “extract 2”.

**Table 1:** Plasmids constructed in this study

**Plasmid**	**Plasmid description**
**YEplacp40Cap**	wt cap sequence including p40promoter was cloned in YEplac181 vector containing 2μm as replication origin and LEU2 as selection marker
**YEplacRepCap**	Entire wt AAV ORFs, rep and cap was cloned in YEplac181 vector containing 2 μm as replication origin and LEU2 as selection marker
**pYESintron Cap**	Cap sequence including intron sequence (without p40) located upstream the start codon of VP1 was cloned downstream Gal1 promoter of pYES2 vector. The selection marker is URA3
**pYESCap**	Entire Cap sequence from start codon of VP1 to the VP3 stop codon was cloned downstream Gal1 promoter of pYES2 vector. The selection marker is URA3
**pYESVP1KM**	VP1 coding region was cloned downstream Gal1 promoter of pYES2 vector. yeast Kozak-like element right upstream VP1 start site. Two mutations in major splice acceptor and a mutation in an out of frame ATG. The selection marker is URA3

In the natural background, the expression of VP proteins is regulated by Rep68 and 78 proteins through the p40 promoter activity and mRNA maturation [23]. The first process is dependent on sequences associated with p5 and p19 promoter elements [24], the second one is achieved when the AAV2 intron element is paired to its natural promoter and extended polyadenylation signal [23]. In order to assess if the expression of Rep could increase the expression of VP1, 2, and 3, we introduced rep and cap gene in their wild type genomic configuration into the yeast vector YEplac181, thus keeping p5, p19, p40, intron and the polyadenylation signal. The resulting plasmid was named YEplacRepCap (Figure 1, scheme “b”). RSY12 cells carrying this construct were grown and collected at two different growth phases, mid-log and late-log phase, and respective protein extracts analyzed by Western blot for Rep and Cap protein expression. All the Rep proteins, namely Rep40, 52, 68 and 78 were expressed and achieved the maximal level at the late-log phase (Figure 2B). Insoluble protein fraction (extract 2) was more abundant in all Rep protein species. To investigate the influence of Rep proteins upon the VP expression, we compared the Western blot results for VPs produced in these cells with the ones from YEplacp40Cap transformants which do not express Rep. VP3 was the only capsid protein detected without a notable difference in its intracellular level and distribution to extracts 1 and 2 (Figure 2C), indicating that Rep proteins had no influence on VP expression in yeast.

### Inducible Yeast Promoter for Regulation of AAV Structural Protein Expression

**Figure 3:**
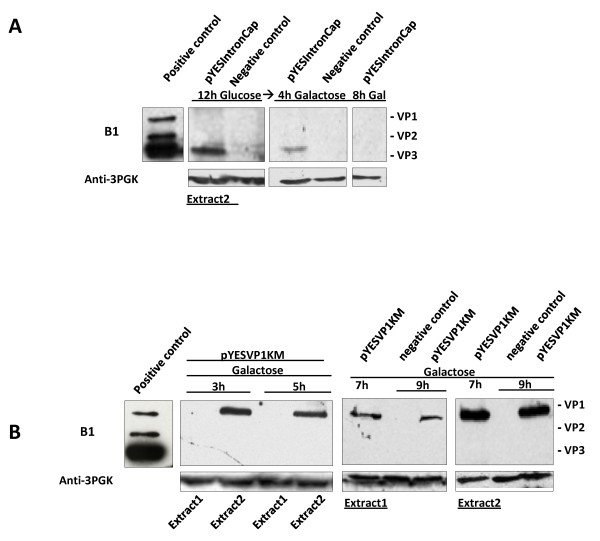
**Expression of AAV2 structural proteins from yeast the galactose-inducible promoter Gal1.** (**A**): pYESIntronCap-transformed cells were first grown for 12 h in glucose and then transferred to galactose medium for 4 and 8 h of induction. Mid-log phase cells were collected at each of these time points and equal amounts of the total cell lysates (~50 μg), were analyzed for Cap protein expression by Western blot using mAb B1. VP3 was the only Cap protein detected and only in the extract 2 (insoluble fraction). Its relative amount was the highest in 12 h glucose samples, diminished after 4 h induction and was no more detectable upon 8 h of galactose induction. (**B**): pYESVP1KM-transformed yeast cells were exposed to galactose for different times, as indicated on the top, and equal amount of corresponding protein (~50 μg) extracts was analyzed for Cap protein expression using mAb B1. The majority of VP1 proteins were found in extract 2 (insoluble fraction). Extracts from cells transformed with empty vector, pYES2 were used as negative controls (−control).

The galactose inducible promoter of the GAL1 gene (pGal1) can be finely regulated by the amount of inducting agent in the growth medium and by varying the induction time [25,26]. Since VP2 was shown to be non essential for infectivity of the AAV virions [27,28], simultaneous expression of VP1 and VP3 in yeast cells was the principal condition for developing yeast-cell based system for production of the wt like AAV-capsids. We made various constructs based on the yeast expression vector pYES2 which contains two potent gene expression regulators, the pGal1 and the *cyc1* terminator (Table 1). To check weather all AAV capsid proteins could be detected simultaneously, these vectors were transformed in yeast and transformants were selected as already reported. Moreover, we made the vector named pYESCap, where the Cap from the VP1 start codon was cloned downstream pGal1 (Figure 1 scheme “c”). Surprisingly, neither VP1 nor VP3 was detected in the extracts from yeast cells carrying the pYESCap ( Figure 1 scheme “c”; data not shown). On the other hand, extracts from yeast cells carrying the vector pYESIntronCap containing the intron sequence upstream VP1 start codon (Figure 1 scheme “d” ), the only protein detected was again VP3 and its highest amount was detected in the extract of the cells grown on glucose (Figure 3A). After the carbon source switch to galactose, some background VP3 level was still observed after 4 h of galactose growth and completely vanished after 8 h in galactose (Figure 3A). The decrease of the amount of VP3 in galactose could not be explained in terms of toxicity since no toxicity-related phenotype (e.g. decreased growth rate or abolished growth) was observed in galactose-grown cells. More likely, carbon source exchange could be quite stressful for the yeast cells.

Studied of AAV protein expression in insect cell system demonstrated that the introduction of some modifications in the coding sequences could improve VP expression [29]. So we made another pYES2-based construct, named pYESVP1KM (Figure 1 scheme “e”) that lacks AAV intron and contains VP1 coding region downstream to pGal1 and yeast Kozak-like element right upstream of VP1 start site. In addition, we introduced two site specific mutations in the VP1 sequence: a mutation in the major AAV splice acceptor site to eliminate the possible splicing from the VP1 mRNA and an out-of-frame ATG mutation in the close proximity to VP1 translational start site to prevent interference with the choice of the right reading frame.

Western blot analysis of protein extracts from pYESVP1KM transformed cells showed that galactose induction caused gradual increase in VP1 protein expression with the highest level achieved after around 7 h of induction (Figure 3B). VP1 was the only capsid protein detected in these cells. The majority of VP1 protein was recovered from insoluble fraction of the total cell proteins (extract 2, Figure 3B).

### Modulation of VP1/VP3 expression pattern as a prerequisite for efficient capsid assembly

**Figure 4:**
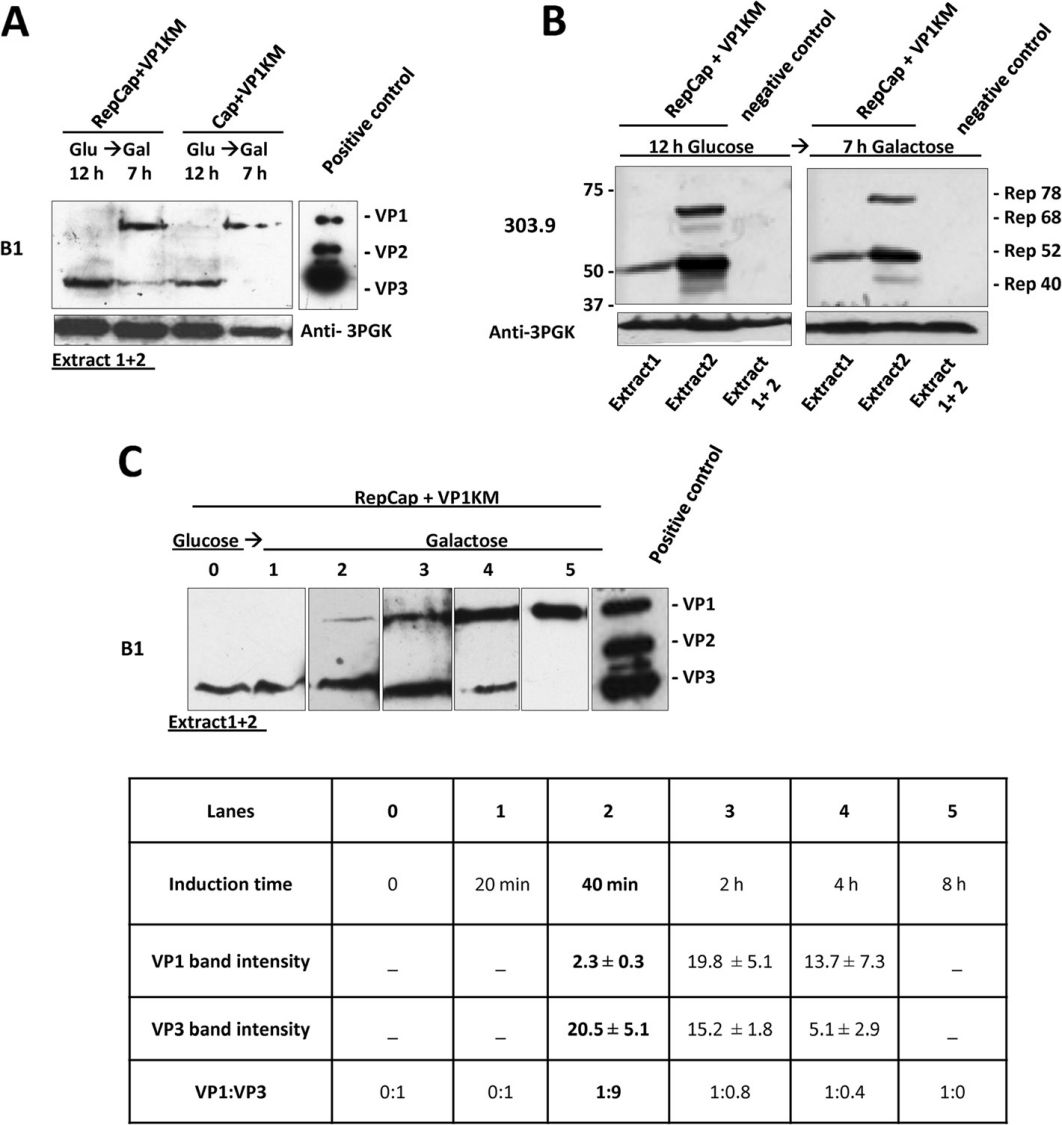
**VP1-VP3 expression pattern in co-transformed yeast clones.** Cells co-transformed with YEplacp40Cap and pYESVP1KM (Cap + VP1KM clone) (**A**) and Rep expressing cells co-transformed with YEplacRepCap and pYESVP1KM (RepCap + VP1KM) (**B**), were grown on glucose and then transferred to galactose for induction. Equal amounts of total cellular proteins (extracts 1 + 2) were analyzed by Western blot, using mAb B1 to detect VP proteins. (**A**)*:* VP3 was detected in both yeast clones after 12 h growth in glucose and it decreased along with VP1 induction upon 7 h in galactose*.* (**B**): Extracts from RepCap + VP1KM clones were analyzed for Rep protein expression before (12 h glucose) and after 7 h galactose growth, using mAb 303.9. Similar amounts and distribution of Rep isoforms to extracts 1/2 were detected in glucose and galactose samples. Extracts from cells co-transformed with empty vectors, YEplac181 and pYES2 were used as -control. (**C**)*: Lanes 0–5*: VP1-VP3 expression pattern in total cell-extracts derived from RepCap + VP1KM clones before induction (“0” time point) and at various times of galactose induction. VP1:VP3 ratios are determined band densitometry and shown in the table below. Numbers represent the density expressed in arbitrary unit detected by the analysis software described in materials and methods. Results are reported as mean of at least three independent experiments ± standard error. The best ratio was obtained after 40 minutes of galactose induction.

**Figure 5:**
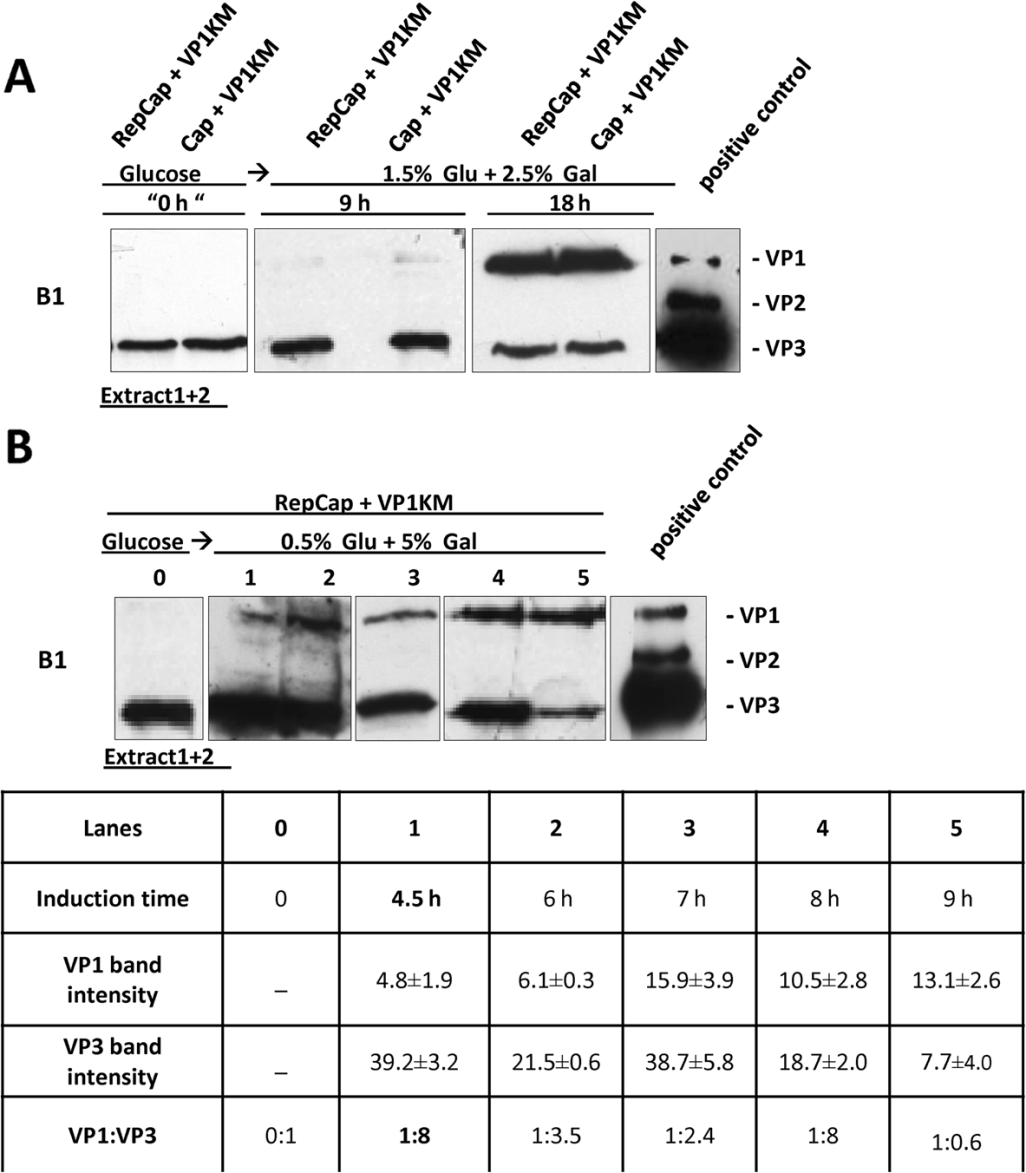
**VP1:VP3 optimization with “low glucose-high galactose” induction strategy.** (**A**) After over-night growth on glucose, YEplacRepCap + pYESVP1KM (RepCap + VP1KM) and YEplacp40Cap + pYESVP1KM (Cap + VP1KM) yeast clones were induced in the presence of high glucose (1.5%) and high galactose (2.5%) concentration. VP expression was analyzed by Western blot at three different time points before induction (“0 h”) and after 9 h and 18 h. There was no significant difference in VP1/VP3 expression pattern between clones and the best ratio (1:9), was detected for 9 h induction time for yeast cells co-transformed with YEplacRepCap and pYESVP1KM (RepCap + VP1KM) . (**B**) After overnight growth on glucose, YEplacRepCap and pYESVP1KM (RepCap + VP1KM) co-transformed yeast cells were induced in the medium containing low glucose (0.5%) and high galactose (5%) concentration. *Lanes 0–5*: VP1-VP3 expression pattern was determined by Western blot analysis before induction (lane1,“0 h”) and after 5 different induction periods (lane 2, 4.5 h; lane 3, 6 h; lane 4, 7 h; lane 5, 8 h, lane 6, 9 h) . VP1:VP3 ratios, calculated by means of band densitometry, are presented in the table below. Numbers represent the density expressed in arbitrary unit detected by the analysis software described in materials and methods. Results are reported as mean of at least three independent experiment ± standard error. The best ratio was obtained after 4.5 h induction in 0.5% glucose + 5% galactose medium (lane 2).

Since we obtained detectable expression of VP3 from the vector YEplacp40Cap, and VP1 from pYESVP1KM, we transformed yeast cells with these plasmids to achieve simultaneous high level expression of AAV capsid proteins. We modulated their relative amount by growing the co-transformed clones, first in glucose and, then, in galactose for different induction times. In parallel, we also tested the clones co-transformed with pYESVP1KM and YEplacRepCap to assess if Rep could affect the VP expression pattern. As expected, Western blot analysis showed that both co-transformed cell clones (Rep positive and Rep negative) produced VP3 protein after growth in glucose, while VP1 expression was induced only after the cell growth in galactose containing medium (Figure 4A). The induction was initially done for 7 h since VP1 was previously shown (Figure 3B) to reach its maximum level at this time point. However, at the end of galactose induction, VP3 protein is not detectable in the cells that do not express Rep (Figure 4A, Cap + VP1KM co-transformed clones) and was hardly detectable in the presence of Rep protein (Figure 4A, RepCap + VP1KM co-transformed clones). This slight difference cannot be only attributed to Rep proteins which, indeed, were efficiently expressed throughout the whole culture (at least Rep78 and 52), both during glucose and galactose growth (Figure 4B). The decrease in the VP3 protein level after the growth in galactose may imply that *de novo* VP3 synthesis is either prevented or reduced when galactose is used as a carbon source, resulting in VP3 “dilution” in the growing cell population. To overcome VP3 decrease and set up the best conditions for production of VP3 and VP1 proteins in the optimal ratio (similar to the one found in the wild type AAV capsids), we gradually decreased the induction time in galactose and analyzed VP1:VP3 ratio at different time points as indicated in the Figure 4C (the densitometry for each induction time is reported in the table below the western blot). VP1 expression was observed already 40 minutes after galactose induction (Figure 4C, lane 2) and increased with time. After 4 hours of induction, gradual increase of VP1 was followed by decrease in the VP3 level (Figure 4C, lane 4), which was no longer detectable after 8 h of induction, when VP1 expression reached its maximum level (Figure 4C, lane 5). The relative VP1:VP3 ratios were calculated from corresponding band intensities at each time point and the values are presented in the Figure 4C. By decreasing induction time to 40 minutes, we obtained the VP1:VP3 ratio of 1:9 (Figure 4C, lane 2). This VP stoichiometry is reported to be in the optimal range to form AAV capsids [18]. Nevertheless, such a short induction time makes the experimental reproducibility very difficult to achieve, so we tried the strategy of “glucose + galactose mixed cultures**”** which enabled fine tuning of VP1:VP3 ratio. When both nutrients are present in high concentration, glucose is used by cells as preferable carbon source. In other words, yeast starts utilizing galactose after the glucose concentration in the medium is completely exhausted [30]. After 12 h growth in 2% glucose medium, cells were transferred to the medium containing 1.5% glucose and 2.5% galactose (named “high glucose, high galactose”) and VP1:VP3 ratios tested at different induction times. The best ratio was reached after 9 h (Figure 5A). After 18 hour induction the VP1 protein level increased and the VP3 decrease. Based on these results, we hypothesized that during the VP1 induction in 5% galactose, glucose should be kept at residual concentration to ensure the constitutive expression of VP3. Therefore, by decreasing the glucose concentration to 0.5%, the optimal VP1:VP3 ratio could be obtained at earlier time points with respect to “high glucose (glu)- high galactose (gal)” conditions. In particular, yeast grown in 2% glucose for 18 hours were transferred in the medium containing 0.5% glucose and 5% galactose, designated as “low glu-high gal” medium. The relative VP1 and VP3 protein level was analyzed by Western blot in extracts from yeast cells collected at different induction time points (Figure 5B). The best VP1:VP3 ratio (1:8) was obtained after 4.5 h of induction (Figure 5B, lane 1, the densitometry for each induction time is reported in the table below the Western blot). When we increased the induction time, VP1 started to accumulate while VP3 decreased leading to a non optimal ratio (Figure 5B, lane 5).

### Assembly and characterization of VLPs

**Figure 6:**
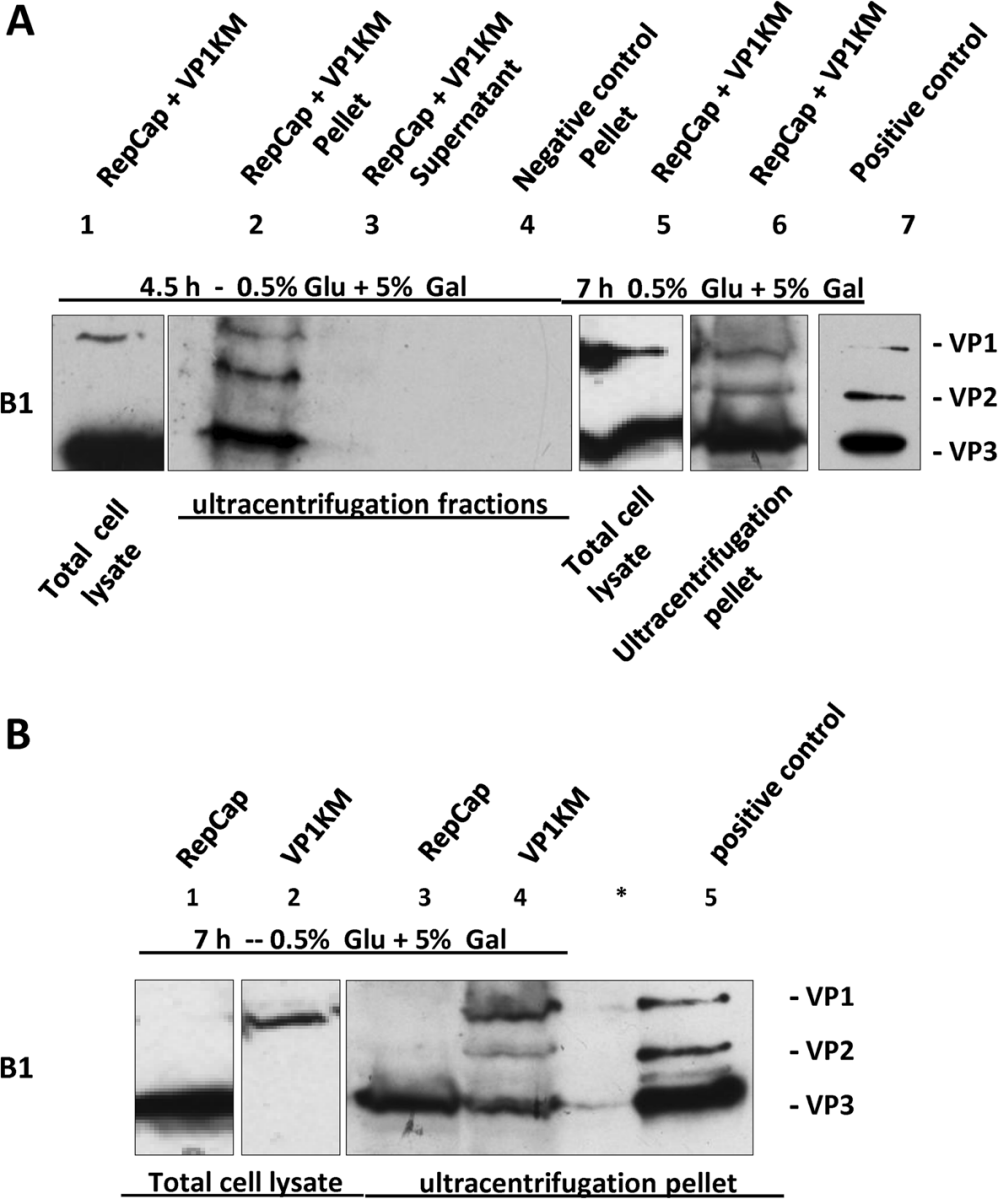
**Concentration of AAV2 capsid like-structures by high-speed ultracentrifugation through 40% sucrose-cushion.** (**A**): YEplacRepCap + pYESVP1KM (RepCap + VP1KM)co-transformed yeast cells induced for 4.5 h (lanes 1–4) or 7 h (lanes 5 and 6) in 0.5% Glu + 5% Gal medium were subjected to a small scale (2 x 10^8^ cells) - protein extraction yielding total cell lysate (lanes 1 and 5) and to a large scale extraction (~400 x 10^8^ cells) (lanes 2, 3, 4 and 6) under non-denaturing conditions yielding the “native extract“ which was subjected to ultracentrifugation. As indicated on the bottom, the total cell lysate (lanes 1 and 5) and the resulting ultracentrifugation fractions, the supernatant (lane 3) and the pellet (lanes 2 and 6), were analyzed for the presence of VP proteins by Western blot. The antibody mAb B1 recognized only VP1 ad VP3 in the total cell lysate and all three VPs in the pellet fraction. The VP ratios in the total cell lysate were 1:8 (lane 1) and 1:3.3 (lane 5). The VP ratios in the pellet resulting from ultracentrifugation were 1:1.2:6.5 (lane 2), 1:3 (lane 6). The pellet obtained by ultracentrifugation of the extracts derived from cells co-transformed with empty vectors, YEplac181 and pYES2, was used as negative control (lane 4). Positive control is loaded in lane 7. (**B**): Cells transformed with YEplacRepCap (RepCap, lane 1 and 3) or with pYESVP1KM (VP1KM, lane 2 and 4) were induced for 7 h in 0.5% glucose + 5% galactose medium. All cells were processed as described in (**A**)*.* VP proteins in the total cell lysates (lanes 1 and 2) and the ultracentrifugation pellet (lanes 3 and 4) were identified by Western blot analysis with mAb B1. The relative VP ratio is 1:0.25:0.9 for sample loaded in lane 4. No proteins were loaded in lanes marked with *. In the lane 5 the positive control was loaded.

To test whether yeast assembled VP1 and VP3 into virus-like particles when these proteins are expressed at the optimal ratio, 3 g of the yeast cell biomass (~400 × 10^8^ cells) carrying the YEplacRepCap and the pYESVP1KM plasmid were lysed and processed under non-denaturing conditions. Cell lysates were subjected to high speed centrifugation in 40% sucrose cushion (as reported in the materials and methods) and the resulting fractions, the pellet (Figure 6A, lane 2) and the supernatant (Figure 6A, lane 3) were analyzed for the presence of VP proteins by Western blot. VPs were found in the pellet (Figure 6A, lane 2) and not in the supernatant fraction (Figure 6A, lane 3). Surprisingly, beside VP1 and VP3, Western blot analysis for the first time revealed the presence of VP2 protein. This result was interpreted in terms of low overall VP2 expression whose concentration in the total cell-lysate (Figure 6A, lane 1) was under Western blot detection limit. To confirm that the ratio of VPs found in the pellet after the ultracentifugation through 40% sucrose cushion, resembled their relative intracellular levels, cells were induced under conditions that yielded VP1 and VP3 in the ratio different from the “optimal” one. For instance, after 7 h of induction in 0.5% glucose + 5% galactose medium, VP1:VP3 in the ultracentrifugation pellet was 1:3 (Figure 6A, lane 6) being almost identical to the ratio in the total cell lysate, 1:3.3 (Figure 6A, lane 5). Again, in the ultracentrifugation pellet is detected also VP2 protein (Figure 6A, lane 6).

Since both constructs used for VP1 and VP3 expression contained unmodified VP2 ORF, comprised in, and in frame with VP1 ORF, we were curious to investigate the origin of VP2 protein. To do this, yeast cells transformed with pYESVP1KM or YEplacRepCap, were induced and processed for ultracentrifugation as double-transformed yeast clones. The two corresponding pellet fractions were analyzed for VP proteins composition by Western blot (Figure 6B, lanes 3 and 4). Total cell extracts were analyzed in parallel to monitor the relative intracellular levels of VPs (Figure 6B, lanes 1 and 2). No VP2 was detected neither in the total protein extract derived from YEplacRepCap transformed cells (Figure 6B, lane 1) nor in the one from the pYESVP1KM clones (Figure 6B, lane 2). The VP composition in the ultracentrifugation pellet derived from YEplacRepCap clones (Figure 6B, lane 3) was identical to the one in its total cell extract: the only protein detectable was VP3. In the pellet from pYESVP1KM–transformed cells, all three VP proteins were detected, suggesting that VP2 and VP3 are synthesized from this construct at low levels (Figure 6B, lane 4). The relative VP1:VP2:VP3 ratio in this pellet was 1: 0.25: 0.9. As expected, the relative amount of VP2 was lower than VP1, whereas the level of VP3 was almost equal to VP1 protein, in divergence with its low overall intracellular level, undetectable by Western blot (Figure 6B, lane 2). This result suggest that even when the concentration of VP was too small for their detection by Western blot, they could assemble into VLPs.

**Figure 7:**
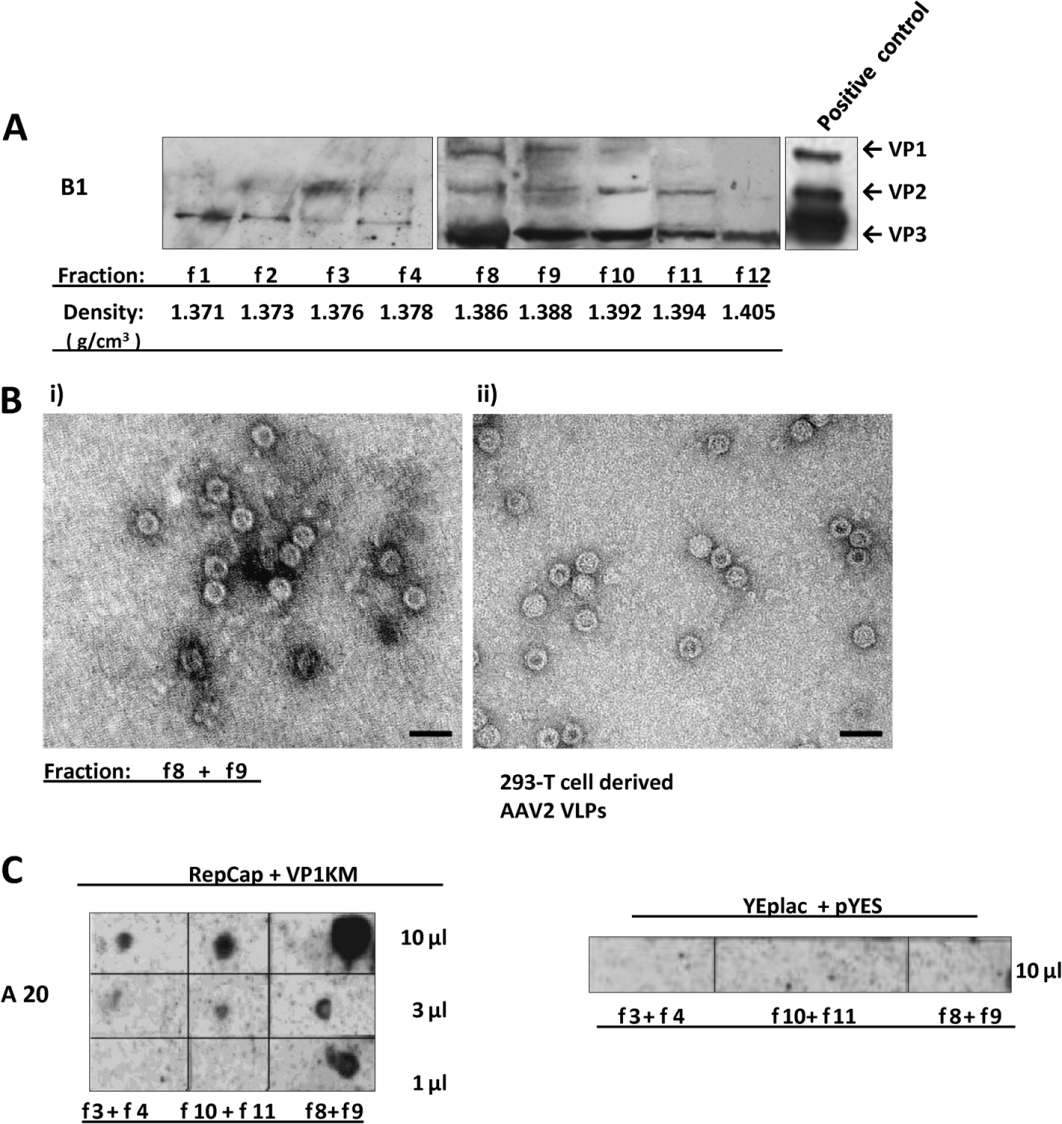
**Isolation of AAV2 capsid like-structures by ultracentrifugation in CsCl-gradient.** Native protein extracts derived from ~ 0.5x10^12^ YEplacRepCap + pYESVP1KM (RepCap + VP1KM)co-transformed yeast cells, induced under optimal conditions, were subjected to 40% sucrose cushion-ultracentrifugation and the pelleted material was further fractionated in CsCl gradient by 48 h,. (**A**): 12 CsCl fractions of increasing densities were recovered and analyzed for the presence of VP proteins by Western blot with mAb B1. Only VP positive fractions are presented. Structures recovered in fractions 8–11 had VP compositions that most closely resembled the one of wt capsids. Denatured 293 T-cell derived AAV2 capsids were used as positive control for defining VPs. (**B**): Fractions of similar densities were united and subjected to TEM analysis. (*i*) Capsid-like structures of ~20 nm size identified in fraction f8 + f9 are shown and compared with 293 T –derived AAV2 empty capsids (*ii*). Scale bar is 40 nm. (**C**): 3 fraction pairs that gave positive results in TEM were spotted on the nitrocellulose membrane in three quantities indicated on the right side bar and analyzed for the presence of AAV capsids with the capsid-specific mAb A20 antibody. The strongest signal (which indicates the greatest number of capsids) was detected in the fraction f8 + f9. As negative control of the assay, the same number of cells co-transformed with empty vectors, YEplac181 and pYES2, were processed as described in (**A**) and the obtained CsCl fractions of the corresponding densities were incubated with A20 antibody. The name of fractions and relative density are indicated.

To better understand if yeast forms VLPs, samples corresponding to 10 g of yeast biomass (YEplacRepCap and pYESVP1KM co-transformed cells grown under “optimal conditions”) were analyzed upon fractionation by high speed ultracentrifugation in CsCl density gradient. After ultracentrifugation, 12 fractions were collected, dialyzed against PBS and analyzed by the Western blot (Figure 7A). In the 1^st^ fraction (of the lowest density), only VP3 protein was detected (Figure 7A, f1). Fractions 2, 3 and 4 (Figure 7A, f2, f3, f4) contained almost equal amounts of VP2 and VP3, but VP1 is below the detection limit. Fractions 8–11 (Figure 7A, f8, f9, f10, f11) contained all three VPs. Only VP3 was detected in f12. Finally, the VP proteins were also detected in the pellet CsCl fraction (f13) (data not shown) and hence not recovered in the other fractions. Importantly, the staining of the nitrocellulose membrane after blotting, showed very low level of VP proteins and no contaminant proteins corresponding to the fractions f8-f12 (data not shown). This result suggest that a very low amount of VLPs was purified. Altogether, our results indicate that yeast is able to assemble AAV VLPs.

To finally demonstrate that yeast supports AAV capsid assembly, small aliquots of fractions of similar densities derived from CsCl gradient fractionation were mixed and analyzed in transmission-electron microscopy (TEM) for the presence of capsid-like structures (Figure 7B). Capsid-like structures were observed in f8-f11 fractions, in the density range of 1.386-1.394 g/cm^3^, containing the three capsid proteins in the relative frequencies similar to the ones of wt AAV-2 capsids. Figure 7B-i shows that yeast-cell derived capsid-like structures observed in fraction f8 combined with f9 (f8 + f9) were morphologically similar to the ones obtained from 293 T cells (Figure 7B-ii, a kind gift from J. Kleinschmidt). The density of capsid positive fractions is not corresponding to the expected ones for empty AAV capsids produced in mammalian cells (1.32-1.35 g/cm^**3**^) [19,31], but is very similar to the density reported for AAV empty particles from insect cells (1.38 g/cm^3^) [32].

To support electron microscopy results, the “VLP-positive” fractions with similar densities were combined resulting in f3 + 4, f10 + f11, f8 + f9 fractions and analyzed in Dot-Blot assay to assess immunoreactivity of yeast-derived particles with anti-capsid A20 antibody. A20 is a widely used monoclonal antibody which specifically binds a conformational AAV-2 capsid epitope and does not recognize native capsid subunits and other assembly intermediates [16][17]. Interestingly, all three fractions showed reactivity to A20 antibody, even f3 + 4 fraction which in western blot analysis showed low level of VP2 and VP3 and VP1 was not detected (Figure 7A). The highest concentration of A20-reactive virus-like particle was observed in f8 + f9 fraction (Figure 7C). This result suggests that the VP proteins assembled in the correct way in yeast.

## Discussion

In line with raising importance of yeast cell-factories in production of VLPs used in vaccinology, diagnostics, sero-epidemiology, nanotechnology and gene transfer, we created the *S. cerevisiae* expression system for studying the permissiveness of its intracellular background to self-assembly of AAV2 capsid proteins in virus-like particles with properties similar to those of AAV capsids produced in mammalian or insect cells. In mammalian cells, the optimal ratio of AAV VPs proteins to obtain high amounts of particles was achieved by placing the rep ORF under a strong promoter and the cap ORF under the control of its natural promoter (p40) [33]. In insect cells, high AAV titers have been obtained using specific *baculovirus* promoters [34].

In order to achieve the best VP protein ratio for the correct assembly in yeast, two requirements has to be satisfied: the simultaneous expression of at least two out of three AAV structural proteins, VP1 and VP3 and the intracellular stoichiometry of these proteins in yeast cells should be similar to that one found in the particles derived from mammalian or insect cells. We constructed one plasmid carrying modified AAV2 Cap ORF under inducible yeast promoter Gal1and the another vector expressing VP3 from the genuine AAV cap gene under its cognate p40 promoter. VP3 protein expression from AAV p40 promoter in yeast cells resembled the expression profile of yeast glycolytic-promoters, characterized by the positive correlation with the cell growth rate during glucose-based growth [35].The best results in terms of “optimal” VP1:VP3 ratio and experimental reproducibility were obtained when glucose was kept at low concentration (0.5%) in the galactose rich (5%) medium. The ratio of VP proteins in cell extracts has been suggested to correlate with VLP composition indicating that the optimal level of proteins is required for the VLP correct assembly [36]. Therefore, after 4.5 hour induction, we have obtained the best VP1:VP3 ratio and we were able to extract VLPs from yeast. Although the CsCl fractions did not have the expected buoyant density, all the VPs proteins were detected and the VLPs were composed of VP1, VP2 and VP3. The buoyant density of infectious AAV particles is reported to range from 1.39 to 1.42 g/cm^3^[31]. Empty or partial empty AAV particles have been shown to have a density corresponding to 1.32 and 1.35 g/cm^3^[31]. On the other hand, AAV empty particles purified from insect cells have a density of 1.38 g/cm^3^ that is not very different from the buoyant density of VLPs from yeast [32]. These light particles appear empty at the electron microscope and have been reported to have either no DNA or DNA of less that genome length; the density of the AAV particles is generally correlated with the size of the encapsidated DNA [37]. We can suppose that higher density of “empty” VLPs from yeast is due to the presence of small yeast DNA fragments inside. This is very difficult to demonstrate since we have a low amount of CsCl fractions because most VLPs remained in the pellet. We have further characterized by electron microscopy VLP fractions with buoyant densities ranging from 1.386 to 1.392 g/cm^3^. Results indicated that the VLPs from yeast are morphologically and immunologically similar (reactivity to A20 antibody) to the particles extracted from human cells (see Figure 7B and C). Our study provides the first experimental evidence that the yeast *Saccharomyces cerevisiae* is able to form AAV VLPs. However, a certain amount of VPLs was not recovered after ultracentifugation. A similar situation was seen in the first baculovirus-insect cell VP-expression system made by Ruffing et al. [38] where the three VPs were expressed from separated ORFs (cDNAs), each one carrying mutations in other two VP-start codons. Moreover, in HeLa cells it has been observed that not assembled VP monomers can associate with cellular structures has been previously documented *in vitro* and [39].

This work demonstrated that *S. cerevisiae* assembles AAV proteins into VLPs and opens new frontiers towards the use of yeast in the rAAV production. Together with our pioneer demonstration of ss rAAV genomes production in this microbial system [8], it leads the future research toward studying yeast permissiveness to packaging of ss rAAV genomes in preformed capsids.

## Competing interest

The authors declare that they have no competing interests.

## Authors’ contributions

AB performed all molecular biology experiments and analyzed the resulting data; TC and LZ carried out the VLP purification; MG analysed the data concerning the CsCl gradient; AS performed the electron microscopy of the VLP; AG conceived the study; AB and TC edited the manuscript. All authors read and approved the final manuscript.
